# Sylvian fissure lipoma with angiomatous component and associated brain malformation: A case report

**Published:** 2013

**Authors:** Shruti Thakur, Ram Gopal Sood, Anupam Jhobta, Sushma Makhaik, CharuSmita Thakur

**Affiliations:** 1 Resident, Department of Radiology, Indira Gandhi Medical College and Hospital, Shimla, Himachal Pradesh, India; 2 Professor, Department of Radiology, Indira Gandhi Medical College and Hospital, Shimla, Himachal Pradesh, India; 3 Associate Professor, Department of Radiology, Indira Gandhi Medical College and Hospital, Shimla, Himachal Pradesh, India; 4 Assistant Professor, Department of Radiology, Indira Gandhi Medical College and Hospital, Shimla, Himachal Pradesh, India; 5 Resident, Department of Radiology, Indira Gandhi Medical College and Hospital, Shimla, Himachal Pradesh, India

**Keywords:** Sylvian Fissure, Angiomatous, Lipoma, Cortical Dysplasia

## Abstract

Intracranial lipomas are congenital malformations. These uncommon lesions have an incidence of 0.1 to 1.7% of all intracranial tumors. Most cases are located at midline and 5% are along the sylvian fissures. If symptomatic, seizures are the most common symptom. These tumors are slow growing and have favorable outcome. We report a case of a 25-year-old man whose CT and MRI revealed a lesion in right sylvian fissure suggesting a lipoma with abnormal vasculature and overlying cortical dysplasia.

## Introduction

Intracranial lipomas are very rare lesions whose development is poorly understood. These lesions are neither hamartomas nor true neoplasms, but are congenital malformations. They may be considered mal-differentiated subarachnoid spaces. Lipomas at sylvian fissure are exceedingly rare. The unusual location of this lipoma prompted the reporting of this case.

## Case Report

**Figure 1:**
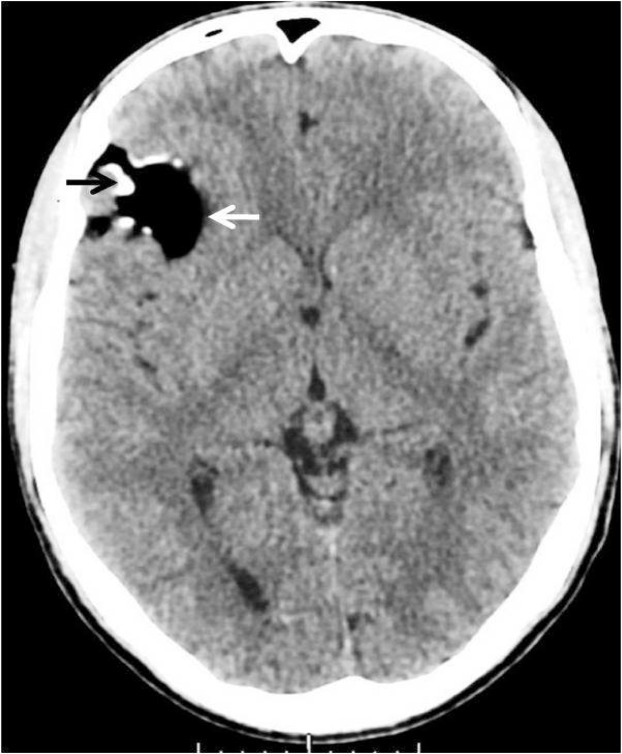
Unenhanced axial CT image shows a large homogeneous right sylvian fissure lipoma with CT value of fat (white arrow). Thick linear calcification is seen at its periphery (black arrow).

**Figure 2:**
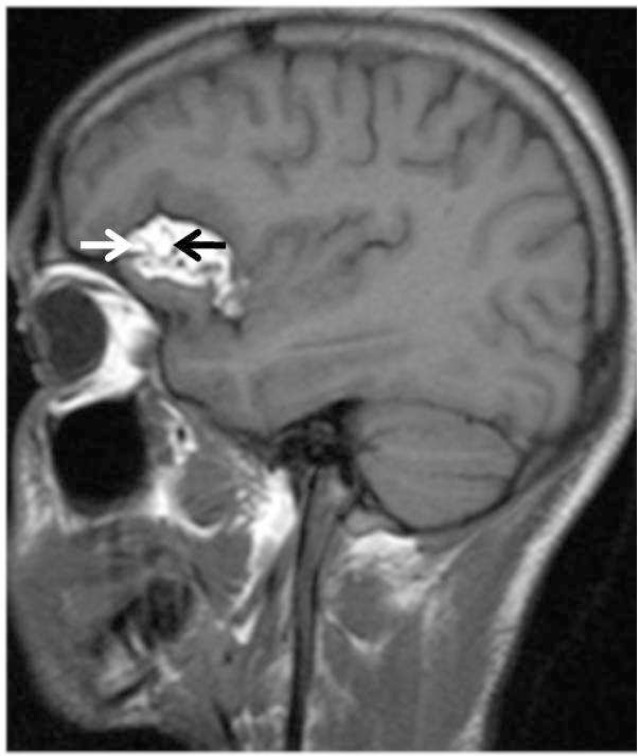
T1-weighted right parasagittal image shows the lesion having typical T1 shortening with bright signal specific of lipomas (white arrow). Multiple linear flow voids are also seen within the lesion (black arrow).

**Figure 3:**
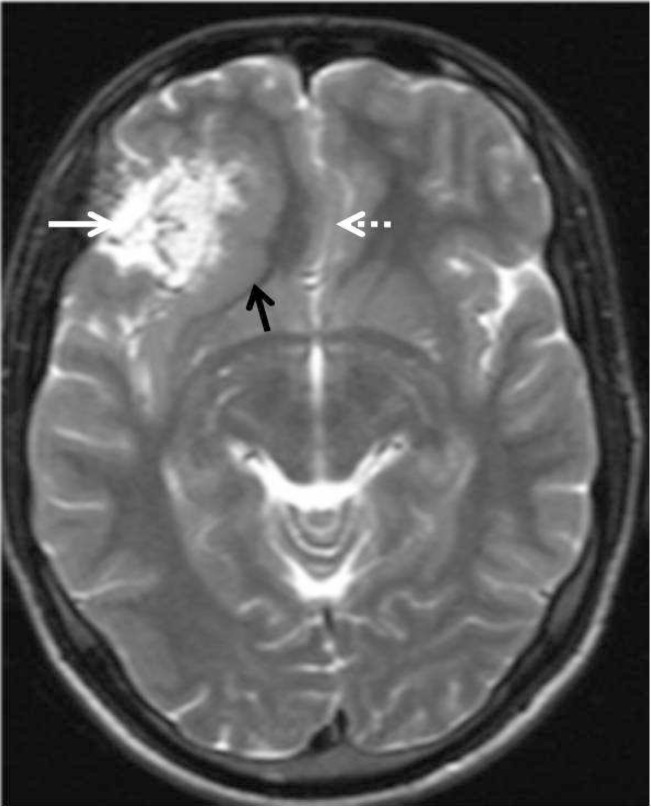
FSE T2-weighted axial image shows hyperintense lesion with flow voids in right sylvian fissure (white arrow) located within a thickened layer of infolding cortex (black arrow). Subfalcine herniation is also seen (dashed white arrow).

**Figure 4:**
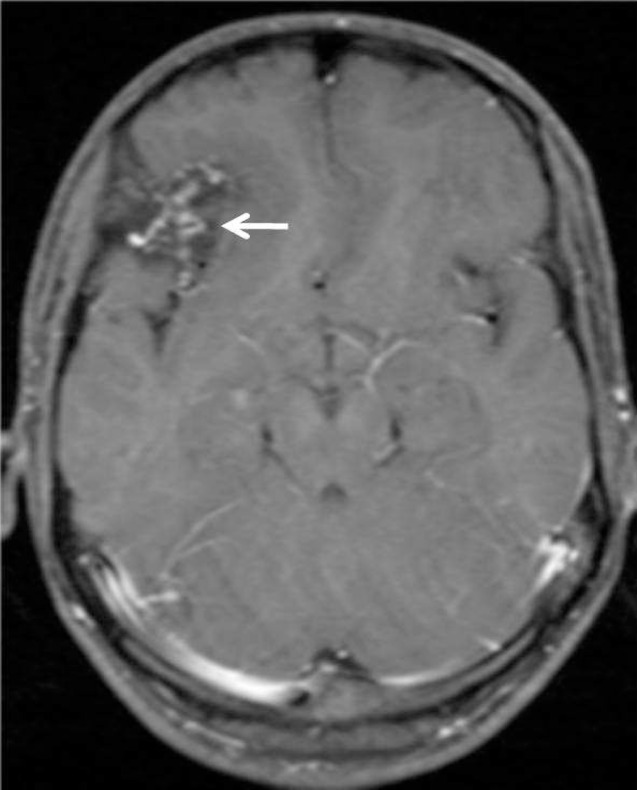
Post contrast fat sat T1- weighted image shows enhancing vessels within the lesion which are extending up to hemispheric surface (white arrow). There is a marked suppression of the bright signal of the lesion.

We report a 25-year-old man who presented with episodic headache for 5 months. He was evaluated because of increasing frequency and severity of the headaches. He had no history of seizures or other significant event. His neurological examination was unremarkable. The patient underwent unenhanced CT of the brain. On CT, a large well defined homogeneous lesion was seen in the right sylvian fissure with attenuation characteristics of fat (-90 to -110 HU). Incomplete peripheral calcification was also seen. The lesion was surrounded by abnormally thick cortex along its medial border (Figure 1). For better evaluation of the lesion, its enhancement pattern and associated cortical malformation, MR imaging was done. On MR, the lesion was hyperintense on both T1 and fast spin echo (FSE) T2-weighted images, and showed multiple flow voids (Figures 2 and 3, respectively). A peripheral hypointense signal was seen corresponding to calcification as seen on CT image. The overlying cortex was thickened with irregular margins. On post contrast fat suppressed T1-weighted images, marked suppression of signal intensity of the lesion was seen consistent with lipoma along with enhancing vasculature within the lesion with its extension to the hemispheric surface (Figure 4). Based on the CT and MR features, the radiological diagnosis of sylvian fissure lipoma with abnormal vasculature and cortical dysplasia was kept. Cerebral angiography was not performed as surgery was not considered. The patient was managed conservatively on medication. Follow-up of patient with MR angiography is planned keeping the possibility of aneurysm formation in the lesion. As our lesion had high intrinsic T1 signal, phase contrast MRA, rather than TOF, would be done.

## Discussion

Intracranial lipomas are uncommon lesions with an incidence of 0.1 to 1.7% of all intracranial tumors.^1^ Most cases are located at midline. 45% of intracranial lipomas are seen along the interhemishere, 25% along quadrigeminal/superior cerebellar cistern, 14% along suprasellar/interpeduncular cistern, 9% along cerebellopontine cistern, and 5% along the sylvian cisterns.^2^ These are congenital malformations that are thought to develop from abnormal persistence and mal-differentiation of the meninx primitiva during the formation of the subarachnoid cisterns. The sylvian fissure is the first sulcus to appear (16-20 weeks of gestation). Redundant meninx primitive can be found in such neural tube flexion sites. Therefore, a lipoma may develop in sylvian fissure. The folding and growth of the surrounding tissues continue until the opercula completely cover the insula to meet in a line which forms the definitive sylvian fissure. Hence, the lipoma may interfere with the development of the adjacent cortical tissue during ongoing formation of the sylvian fissure.^3^ Varying degrees of associated brain malformations are seen in 55% of the lesions, and 36% of the lesions show traversing intracranial vessels and nerves through them.^2^ The associated CNS malformations are agenesis or dysgenesis of the corpus callosum, absence of septum pellucidum, cranium bifidum, spina bifida, encephalocele, myelomeningocele, hypoplasia of vermis, and cortical dysplasias.^3^ The possible explanations of abnormal vasculature are manifold. One explanation of aneurysm formation is congenital structural deficiency, as the artery shares the same malformative origin with lipoma. Another explanation is that lipoma might secrete some tumoral factors which weaken the arterial wall. A third explanation may be that lipoma itself causes degeneration of arterial wall by interfering with arterial nutrition. The cerebral artery does not have vasa vasorum and derives its nutrition from CSF by diffusion. Some lipomas may show amorphous calcification. The bony components are foci of osseous metaplasia. In such a case, the diagnosis of osteolipoma is made that shows ossification histopathologically.^4^

Sylvian fissure lipomas are the least common of all the intracranial lipomas with only 13 cases reported until 2007 and the angiomatous variety still rarer.^1^ Rokitansky reported the first case of intracranial lipoma as an accidental finding at autopsy in 1856. The first case was diagnosed in a living patient in 1939 by Sosman. In 1983, Hatashita et al. reported the first case of sylvian fissure lipoma, in which the lesion was partially removed and diagnosis surgically verified.^5^ Intracranial lipomas are often asymptomatic. The most common symptom is seizures. However, the patient may also present with headache, emotional instability, hemiparesis or intellectual disorder.^6^ Clinical progress in the form of onset and worsening of symptoms is thought to be caused by tumor growth. If the lesion is causing no sign or symptoms and is diagnosed incidentally, the patient should be managed expectantly. Surgical intervention is indicated if the lesions cause symptoms. These lesions are attached inadvertently to the surrounding structures. An attempt to totally resect the lesion may injure adjacent brain. Therefore, in most cases, only partial removal is contemplated. These tumors are slow growing with favorable outcome. Therefore, the primary aim of surgery is adequate decompression.

On CT, the lipomas appear as well-defined homogeneous lesions with CT attenuation characteristic of fat. Calcification may be seen. There is no contrast enhancement except in the angiomatous variety, in which enhancing vessels may be seen within the lesion. On MRI, these lesions demonstrate fat signal with a short T1 and T2 relaxation times; that is, bright homogeneous signal on T1-weighted images and intermediate signal on T2-weighted images. Moreover, the signal is bright on FSE/TSE T2 sequences. Intracranial lipomas need to be differentiated from other intracranial fat containing lesions. Dermoids are less homogeneous and have higher attenuation values. Furthermore, with fat suppressed sequences, lipomas exhibit more signal intensity suppression than do dermoids. Teratomas are heterogeneous and may show contrast enhancement.

## Conclusion

Sylvian fissure lipomas are rare lesions, and with angiomatous component and cortical dysplasia, these lesions are even rarer. Our patient is managed without surgery with the diagnosis made on radiological grounds. If the lesion becomes symptomatic or diagnosis is in question, surgery will become mandatory.

## References

[1] Chao SC, Shen CC, Cheng WY (2008). Microsurgical removal of sylvian fissure lipoma with pterion keyhole approach-case report and review of the literature. Surg Neurol.

[2] Truwit CL, Barkovich AJ (1990). Pathogenesis of intracranial lipoma: an MR study in 42 patients. AJR Am J Roentgenol.

[3] Saatci I, Aslan C, Renda Y, Besim A (2000). Parietal lipoma associated with cortical dysplasia and abnormal vasculature: case report and review of the literature. AJNR Am J Neuroradiol.

[4] Park YS, Kwon JT, Park US (2010). Interhemispheric osteolipoma with agenesis of the corpus callosum. J Korean Neurosurg Soc.

[5] Sarioglu AC, Kaynar MY, Hanci M, Uzan M (1999). Sylvian fissure lipomas: case reports and review of the literature. Br J Neurosurg.

[6] Rahalkar AM, Rahalkar MD (2006). Case report: 2 cases of lipoma of corpus callosum (LoCC) associated with lipoma of choroid plexus (LoCP). Indian J Radiol Imaging.

